# Sustainable Printed Electrochemical Platforms for Greener Analytics

**DOI:** 10.3389/fchem.2020.00644

**Published:** 2020-07-30

**Authors:** Patrick Severin Sfragano, Serena Laschi, Ilaria Palchetti

**Affiliations:** ^1^Department of Chemistry “Ugo Schiff”, University of Florence, Sesto Fiorentino, Italy; ^2^Ecobioservices and Researches SrL, Florence, Italy

**Keywords:** printed sensors, electrochemical, green analytics, polylactic acid, silk proteins, cellulose, biochar

## Abstract

The development of miniaturized electrochemical platforms holds considerable importance for the *in situ* analytical monitoring of clinical, environmental, food, and forensic samples. However, it is crucial to pay attention to the sustainability of materials chosen to fabricate these devices, in order to decrease the amount and the impact of waste coming from their production and use. In the framework of a circular economy and an environmental footprint reduction, the electrochemical sensor production technology must discover the potentiality of innovative approaches based on techniques and materials that can satisfy the needs of environmental-friendly and greener analytics. The aim of this review is to describe some of the printing technologies most used for sensor production, including screen-printing, inkjet-printing, and 3D-printing, and the low-impact materials that are recently proposed for these techniques, such as polylactic acid, cellulose, silk proteins, biochar.

## Introduction

Electrochemical sensors and biosensors are routinely used for healthcare, environmental, food quality control, forensic, and security applications.

Thin film and thick film technologies are the main techniques for electrochemical sensor production. Thin film technologies, i.e., lithographic methods, allow the development of reproducible, high performance devices, but unfortunately, fabrication costs are generally high. Thick film technologies, such as printing methods, can significantly lower the production costs while allowing mass-production of devices with desired reproducibility, and have attracted significant interest over the years for the fabrication of sensors (Turner, 2013).

**Figure 1:**
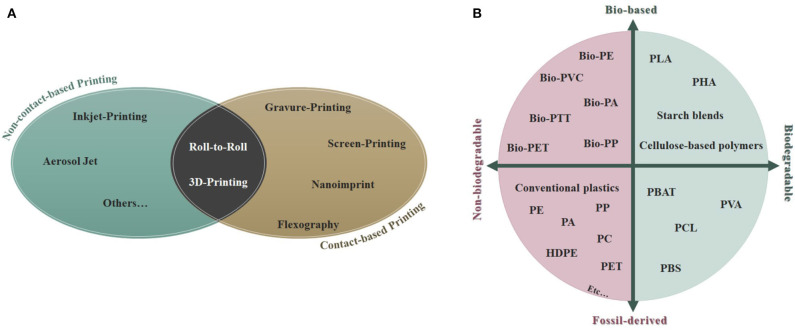
**(A)** Classification of the main contact- and non-contact based printing technologies; **(B)** Schematic classification of the different types of plastics and bioplastics. PLA, polylactic acid; PHA, polyhydroxyalkanoate; PVA, polyvinyl alcohol; PBAT, polybutylene adipate terephthalate; PCL, polycaprolactone; PBS, polybutylene succinate; PE, polyethylene; PP, polypropylene; PA, polyamide; PC, polycarbonate; HDPE, high-density polyethylene; PET, polyethylene terephthalate; PTT, polytrimethylene terephthalate; PVC, polyvinyl chloride.

Printing techniques for sensor production could be broadly grouped in: (a) contact-based techniques (i.e., screen-printing, gravure-printing, pad-printing, stamp-assisted printing, flexographic-printing, etc.) where there is a physical contact between the printing medium with the designed structures of the target substrate, and (b) non-contact printing processes (such as inkjet-printing, aerosol-jet printing, etc.) (Khan et al., 2019). Roll-to-roll manufacturing and three-dimensional (3D)-printing are novel technologies that stand in between of the two aforementioned categories (Figure 1A).

Screen-printing is a contact-based technique and one of the most matured and common technologies for printed electronics and large-scale, disposable sensor production. Many examples of different formats of such sensors are nowadays reported in literature (Palchetti et al., 2000; Centi et al., 2007; Bettazzi et al., 2013; Voccia et al., 2017).

The setup consists of a variety of elements, including screen, squeegee, and substrate. A mounted frame carries a tightly stretched mesh made of polyester, stainless steel, or nylon, structured in a particular way to block the unnecessary openings in the mesh for the reproduction of the desired pattern. The squeegee, with a certain pressure and moving at a given velocity, presses the paste through the openings in the screen, thus transferring it onto the substrate beneath it. The pattern can be realized with different electrode geometries and on a wide range of substrates, such as paper, plastic, ceramics, glass, and metal. In addition, it allows the obtainment of thick layers ranging from below 1 μm up to several hundreds of micrometers with a single pass, often not achievable with other techniques.

Screen-printing is characterized by a large availability of pastes (i.e., conductive, semiconductive, and dielectric pastes are commercially available). The viscosity required highly depends on the mesh aperture width and the pressure applied (Kim et al., 2017). This aspect also affects the printing speed, which is the main disadvantage of this technique together with the necessity to study and correctly adapt a variety of parameters in order to achieve a high precision.

Inkjet-printing is a widespread non-contact technique that consists in the direct deposition of pastes through a micrometric nozzle head that relies on piezoelectric, thermal or electrohydrodynamic actuation for droplet ejection. The paste should possess a good chemical stability and solubility in common solvents, alongside a low temperature processing. The viscosity and the surface tension play key roles in this technique, as well as the choice of the appropriate concentration and solvent system (which has to be removed after printing), in order to prevent the formation of clogs in the nozzles.

Inkjet-printing is an entirely digital technique: the print pattern is a computer-aided design (CAD) modifiable with little cost impact and sent directly to the printer (Tortorich et al., 2018). Moreover, as opposed to screen-printing where large amounts of paste are required, inkjet-printing uses very little amount of material, which is always a good advantage in terms of eco-sustainability. Furthermore, this technique reduces the amount of dangerous and environmentally-sensitive wastes (Sui and Zorman, 2020).

The most common conducting pastes are based on gold (Au), silver (Ag), platinum (Pt), and carbon nanomaterials (i.e., graphene, carbon nanotubes, carbon powder) (Moya et al., 2017). Inkjet-printing is suitable for printing on rigid substrates, but it is particularly well-suited to flexible substrates (Al-Halhouli et al., 2018), which are progressively more demanded in many fields (e.g., biomedical, wearable) and nowadays are mainly made of plastic materials (e.g., polyethylene terephthalate, PET). As for screen-printing, the sintering process of the conductive materials is commonly accomplished by thermal annealing. However, low-temperature methods, such as chemical treatment and photonic radiation, can also be performed.

The main challenging factors of inkjet-printing are the slow speed, due to limited number of nozzles, and the strict rheological conditions that inks need to meet in order to avoid nozzle clogging. In addition, there is a deficiency of commercial inks, which are also expensive and have very limited shelf-life. For these reasons, inkjet-printing finds some obstacles in becoming an industrial production technique for printed electronics (Khan et al., 2015). Moreover, fully inkjet-printed electrochemical sensors are rather difficult to obtain due to difficulties to print all the components required, such as the sensing layer (Moya et al., 2017). Therefore, inkjet-printing is often combined with other manufacturing techniques (e.g., screen-printing).

3D-printing, known as additive manufacturing, is a cutting-edge family of techniques that fabricates 3D components by stacking layers of materials according to a digital 3D model of the intended object.

Compared to other printing techniques, 3D-printing is more compatible with a vast array of substrates, from rigid supports to flexible films, and provides a great freedom in terms of choice of substrate materials and object design (Fan et al., 2019). In addition, it is presented as a versatile and inexpensive technology, due to the introduction of cheaper, simpler, and desktop printing machines. The materials used are often filaments of polymers, metals, composites, and ceramics that get extruded through a heated nozzle. Composites of thermoplastics and carbon-based materials (i.e., graphene, graphite, carbon black, carbon nanotubes) are used as conductive materials together with the insulating nature of thermoplastics filaments (e.g., acrylonitrile butadiene styrene, ABS) (Wei et al., 2015; Katic et al., 2019; Foster et al., 2020; Kalinke et al., 2020).

In electrochemistry, 3D-printing is being increasingly considered since it can be employed to obtain both the conductive parts and the substrate of a sensor, with desired size, shape, configuration, and material composition (Ambrosi and Pumera, 2016).

A variety of different 3D-printing technologies exist and involve the creation of three-dimensional objects using: material and binder jetting, material extrusion, sheet lamination, photopolymerization, powder bed fusion, etc. (Xu et al., 2017). Among the 3D-printing technologies, the Fused Deposition Modeling (FDM) is widely used for the fabrication of electrochemical systems (Fan et al., 2019; Katseli et al., 2019), thanks to its easy processability and the low-cost of printers.

However, 3D-printing is still finding its way in various fields of analytical chemistry and only a few applications on electrochemical sensing have appeared in literature. To this end, few review papers and research articles (Rymansaib et al., 2016; Xu et al., 2017; Cardoso et al., 2020) have reported applications of this technology in sensor fabrication.

Roll-to-roll (R2R) methods are drawing increasing attention in recent years within the field of (macro) electronic devices, due to the need for extremely fast and relatively inexpensive large-scale manufacture of electronic devices, characterized by thin films patterned over large substrate areas (Bariya et al., 2018).

The R2R production line is composed of a number of rollers over which the web (flexible substrates) passes with controlled tension, while the deposition of diverse materials occurs. This process is thus composed of a variety of steps, including the unwinding of the web roll, pre-treatment (e.g., thermal curing), printing of the device nanolayers, encapsulation, and to conclude the cycle, web roll winding.

R2R-fabrication is quite attractive for organic/polymer-based thin film devices (e.g., PET) and it has found applications in the development of solar cells, light-emitting diodes, and more recently, sensors (Khan et al., 2015). Among these techniques, Roll-to-roll gravure-printing has recently gained increased interest as a production process for printing electronic devices (Noh et al., 2010); nevertheless, flexography, rotary screen-printing, and nanoimprint techniques are also favorable candidates for Roll-to-roll configurations.

Several papers have described the technical details of the different printing techniques (Khan et al., 2015; Ambrosi and Pumera, 2016). The aim of this review is to discuss the recent literature on sustainable functional materials for electrochemical sensor development by using printing procedures. Sustainable materials, i.e., materials allowing less release of hazardous products in the environment with a reduction of wastes and the re-use of waste materials (Chen et al., 2020), are reviewed in terms of printing substrates and of components of printing pastes and inks.

## Novel Materials for Printing

A printed electrochemical sensor is mainly composed of layers of conductive as well as dielectric pastes printed on an inert substrate. The pastes are mainly based on functional materials consisting of micro- and nanoparticles colloidal suspension, containing additives, solvent, and dispersing agent or binders. Non-degradable polymers are frequently used as printing substrate (i.e., polyester) and binders (i.e., polyester, acrylic, polysulfone-based resins). Thus, in order to obtain a sustainable printed electrochemical platform, it is necessary to use greener materials for the substrate and the pastes.

### Polylactic Acid-Based Materials

A promising and burgeoning alternative to non-degradable commodity polymers seems to be the utilization of biodegradable plastics, already employed in tissue engineering and medicine (Haider et al., 2019). Among these biodegradable polymers, the most produced nowadays are polyhydroxyalkonates (abbreviated with PHAs), which account for 6% of the global production capacity, polybutylene succinate, and polybutylene adipate terephthalate (PBS and PBAT, respectively; 23% of total production) and polylactic acid (PLA; 24%) (Haider et al., 2019), (Figure 1B). PLA is obtained from 100% renewable sources (i.e., sugar beets and corn) (Mühl and Beyer, 2014). It presents a good biodegradability as well as an excellent biocompatibility, making it a great polymer for applications in the medical/biomedical field and for wearable sensors (Khan et al., 2019).

Different types of PLA substrates are commercially available and the literature has been therefore enriched by various examples of applications of PLA as substrate for the fabrication of electrodes (Quintero et al., 2014; Mattana et al., 2015; Fan et al., 2019) by different printing techniques and as component of conductive pastes and filaments.

**Figure 2:**
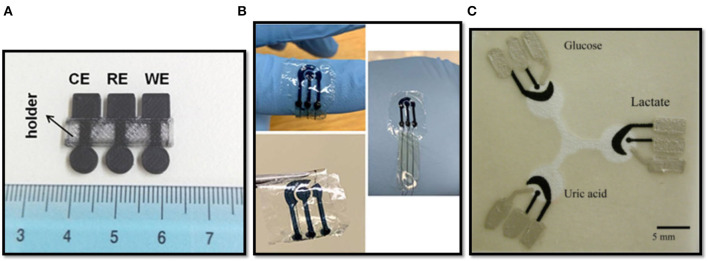
**(A)** 3D-printed 3-electrode integrated device made with PLA filaments, reprinted with permission from Katseli et al. (2019); **(B)** PEDOT:PSS patterns on flexible silk fibroin sheets, reprinted with permission from Xu et al. (2019); **(C)** Paper-based microfluidic electrochemical chip, reprinted with permission from Dungchai et al. (2009).

Recently, the development of 3D-printed electrochemical sensors based wholly on PLA was achieved by Katseli et al. (2019): using a non-conductive filament of PLA to build the insulating support and a conductive PLA filament (i.e., a carbon-loaded PLA filament, C-PLA) to obtain electrodes, a versatile electrochemical sensing platform was achieved (Figure 2A). Electrochemical characterization was performed by linear sweep voltammetry in different media at different pH values. The sensor has shown a wide range of operational potential, comparable with that of conventional carbon-electrodes. The determination of mercury by Square Wave Anodic Stripping Voltammetry (SW-ASV) in 0.1 M HCl was carried out after a preconcentration of 360 s, obtaining a limit of detection (LOD) of 1.9 μg/L. Differential pulse voltammetry (DPV) was used for the determination of caffeine. Well-resolved DPV-peaks were obtained in H_2_SO_4_ (0.2 M) as supporting electrolyte. A linear correlation between peak currents and concentration (*R*^2^ = 0.998) was reported, with a LOD of 1.8 mg/L. Furthermore, the sensor was applied for the determination of glucose, by developing a biosensor containing the enzyme glucose oxidase (GOx). GOx was successfully immobilized on the surface of the working electrode. The determination of the H_2_O_2_ resulting from the enzymatic oxidation of glucose was performed by chronoamperometric analysis at −0.6 V vs. C-PLA reference electrode (Katseli et al., 2019). A graphene-based PLA (G-PLA) working electrode was assembled by 3D-printing for the determination of phenol compounds (Cardoso et al., 2018). In another report, an electrode was modified by the electrodeposition of gold for catechol determination (O'Neil et al., 2019). G-PLA disk- and ring-shaped electrodes were used for the determination of ascorbic acid and picric acid (Manzanares Palenzuela et al., 2018).

More recently, carbon black/polylactic acid 3D-printed electrodes have been used for quality control testing of fuel bioethanol, monitoring the levels of Cu(II) by SW-ASV (João et al., 2020). PLA is soluble in many organic solvents, but insoluble in alkanes (i.e., *n*-dodecane). Thus organic solvents are generally used to remove or reduce the potential protective layer created by PLA when utilized together with a conductive material, in order to achieve electrodes with a better conductivity (dos Santos et al., 2019; Gusmão et al., 2019; Richter et al., 2019; Wirth et al., 2019). In this context, the work of Kalinke et al. (2020) showed a considerable improvement of the electrochemical properties of a 3D-printed electrode made of G-PLA when the graphene nanoribbon structures were exposed through the reduction of the protective layer of PLA, accomplished by solvents or saponification (Kalinke et al., 2020). An electrochemical characterization of such device was performed by cyclic voltammetry (CV). DPV and SWV were used for the determination of dopamine in phosphate buffer (pH 6.0), in serum and in synthetic urine. Both DPV and square wave voltammetry (SWV) exhibited two linear regions at different concentrations, with a LOD of 2.17 and 1.67 μmol/L respectively. Dopamine concentration was also evaluated in human or human-like biological fluids, characterized by the interfering co-presence of ascorbic acid and uric acid, obtaining a LOD of 1.25 μmol/L *via* DPV. Moreover, a good repeatability and reproducibility [relative standard deviation (RSD) of 2.67 and 7.14%, respectively] have been achieved. Different electrochemical treatments have also been reported in literature for removing non-conductive polymer layers (Rocha et al., 2020).

A silver pseudo-reference electrode produced by 3D-printing has been recently electrochemically characterized by Pumera's group (Rohaizad et al., 2019).

### Silk Proteins

Silk proteins, such as natural fibroin, have been largely utilized for a variety of applications in several fields and have been harnessed as substrates for conductive materials, especially in applications where a good flexibility is needed.

Pal et al. (2016) showed a biocompatible and water-based conductive paste based on natural and photoreactive silk proteins, patterned onto crosslinked silk fibroin sheets, in order to achieve a fully biodegradable electrochemical sensor. In particular, in their work they have characterized mechanically and electrochemically a sericin/poly(3,4-ethylenedioxythiophene)-poly(styrenesulfonate) (PEDOT-PSS) device. An electrochemical sensing characterization was performed, carrying out the determination of ascorbic acid (AA) and dopamine in phosphate buffer saline (PBS) solutions. A LOD of 15.21 and 15.47 μM were obtained for AA and dopamine, respectively. Furthermore, the determination of glucose by the immobilization of GOx in the above-mentioned paste was accomplished with a LOD of 1.16 mM (Pal et al., 2016). Likewise, Xu et al. (2019) compared the chronoamperometric response of such silk-proteins-based device (Figure 2B) with a conventional one for the determination of AA, obtaining a LOD of 49.2 μM for the sericin/PEDOT-PSS-based device and 50.2 μM for the conventional one.

### Cellulose Based Materials

Cellulose is a material suitable to form both biodegradable substrates and paste formulations for printed electrochemical sensors.

Cellulose fiber is the main constituent of paper. Paper-based microfluidics, namely the fluid transportation through spontaneous capillary action of paper, combined with electrochemical techniques, has attracted intensive research attention over the years. With its particular fibrous and porous structure, paper allows fluids (e.g., water) to deliver analytes to electrode surfaces without the need of pumps or other external pressure control systems, thus resulting an ideal substrate for “miniaturizable,” portable and disposable electrochemical devices, characterized by a low cost, a high flexibility, a high sensitivity, and the ability to perform a variety of measurements (Shen et al., 2020). Whiteside's group pioneered the field of paper-based analytical devices (Martinez et al., 2007). Dungchai et al. (2009) reported one of the first paper based electrochemical sensors (Figure 2C). The simultaneous determination of glucose, lactate, and uric acid in biological fluids was demonstrated by modifying the electrode surface with GOx, Lactate oxidase and uricase, respectively. H_2_O_2_ produced by the enzymes was measured using chronoamperometry at 0 V vs. on-chip Ag/AgCl reference electrode (Dungchai et al., 2009).

Different types of papers can be used, depending on analytical requirements (Cinti et al., 2019). Since the field of paper-based printed electrochemical sensor is quite vast, we would like to invite the reader to refer to detailed review papers inherent this topic (Martinez et al., 2010; Hu et al., 2014; Medina-Sánchez et al., 2015; Jia et al., 2016; Chouler et al., 2018; Gebretsadik et al., 2019; Smith et al., 2019). Recently, bacterial-derived cellulose substrates have been proposed for the development of screen-printed sensors for the determination of lactate (Gomes et al., 2020) and heavy metals in sweat (Silva et al., 2020), respectively.

Cellulose-based materials are also interesting materials for the production of pastes. Cellulose nanofibrils and cellulose nanocrystals modified with conductive polymers (Hoeng et al., 2017; Latonen et al., 2017), metal nanoparticles and carbon nanomaterials have been proposed as water-based pastes for screen-printing and ink-jet technologies (Couto et al., 2016; El Baradai et al., 2016). Moreover, ethyl cellulose (Hatala et al., 2019) and carboxymethyl cellulose (Barras et al., 2017), water-soluble derivatives of cellulose, are often used as dispersing agent.

### Other Materials

A promising role may be played by conductive/semiconductive engineered materials produced from bioresources and recycling residues and waste from industrial processes. Carbon black and biochar are interesting examples. Biochar is the carbon-rich material produced from organic feedstock such as agricultural wastes and municipal solid waste in limited oxygen atmosphere and under certain thermal combustion. Some examples of using biochar for electrochemical sensing are reported in literature (de Almeida et al., 2020). Other carbonaceous materials from biomass have already been used for electrode production (Ferreira et al., 2018; João et al., 2020; Rocha et al., 2020).

Li et al. (2018) proposed electrically conductive and mechanically stable carbon nanofiber aerogels made from wood-derived nanofibrillated cellulose. Minakshi et al. (2020) presented a hybrid electrochemical device produced by calcinated eggshell obtained from biowaste with a mixed binary metal oxide (NiO/Co_3_O_4_) to obtain, respectively, the anode and the cathode of such device.

Finally, some conductive polymers, such as polydopamine, have emerged in the production of semiconductor materials since they are efficient and less toxic alternatives to certain kinds of inorganic semiconductors.

## Conclusions and Future Perspectives

In this review, we have summarized the recent materials for a greener sensor production, also describing the main techniques for sensor fabrication. Nowadays, paper-based technology is a mature technology and a plethora of analytical applications have been already reported. Moreover, PLA-based devices seem particularly promising as alternative to classical non-biodegradable polymer-based tools. Similarly, carbonaceous and other materials derived from biomass and industrial wastes seem particularly interesting for obtaining reliable electrode materials.

A better understanding of mechanical and electrochemical properties of these materials might lead to the development of next-generation pastes and substrates for sustainable electrochemical sensor production.

## Author Contributions

The manuscript was written through the contribution of all authors. All authors have approved the final version of the manuscript.

## Conflict of Interest

SL was employed by the company Ecobioservices and Researches SrL. The remaining authors declare that the research was conducted in the absence of any commercial or financial relationships that could be construed as a potential conflict of interest.
